# 2-Benzyl-3-hy­droxy-3-methyl-2,3-dihydro-1*H*-isoindol-1-one

**DOI:** 10.1107/S1600536812021575

**Published:** 2012-05-19

**Authors:** Hong-Yao Wang, Jing-Kui Yang

**Affiliations:** a College of Chemistry and Chemical Engineering, Graduate University of Chinese Academy of Sciences, Beijing 100049, People’s Republic of China

## Abstract

In the title compound, C_16_H_15_NO_2_, the isoindoline ring system is approximately planar (mean deviation = 0.0186 Å) and makes a dihedral angle of 61.91 (4)° with the phenyl ring. In the crystal, mol­ecules form inversion dimers *via* pairs of O—H⋯O hydrogen bonds.

## Related literature

For background to the synthesis of the title compound, see: Griffiths *et al.* (1983 ▶); For its applications in synthesis, see: Winn & Zaugg (1968 ▶); Katsuhiko *et al.* (2006 ▶). For related structures, see: Wang *et al.* (2008 ▶); Orzeszko *et al.* (1998 ▶); Liu *et al.* (2009 ▶); Rosamilia *et al.* (2002 ▶).
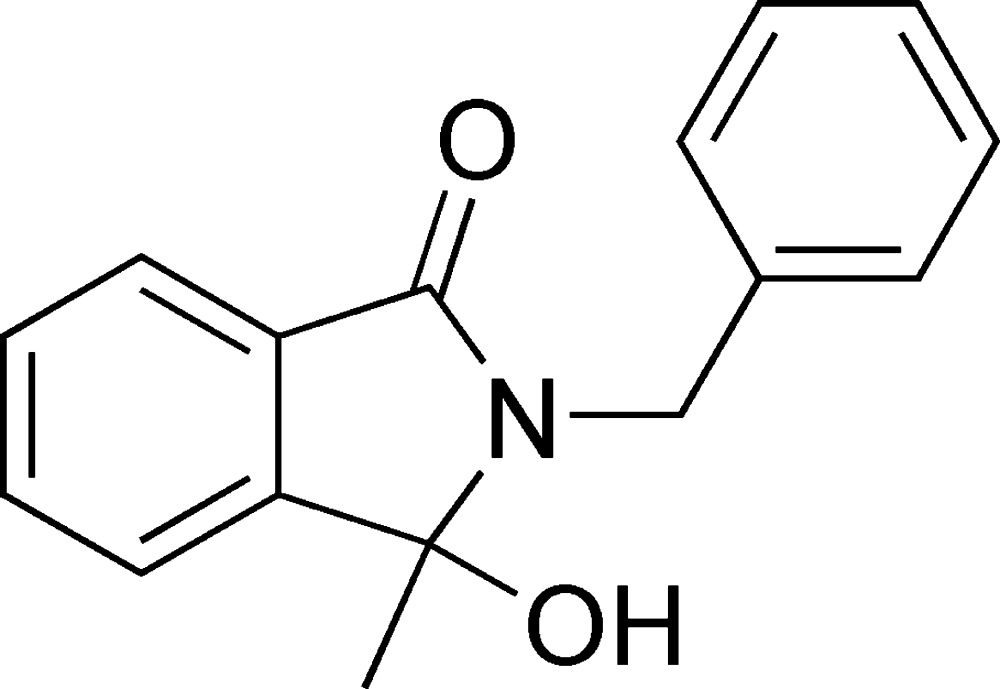


## Experimental

### 

#### Crystal data

C_16_H_15_NO_2_
*M* *_r_* = 253.29
Monoclinic, 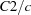

*a* = 11.093 (4) Å
*b* = 11.604 (4) Å
*c* = 21.226 (7) Å
β = 101.777 (5)°
*V* = 2674.7 (15) Å^3^
*Z* = 8
Mo *K*α radiation
μ = 0.08 mm^−1^
*T* = 153 K
0.47 × 0.34 × 0.23 mm

#### Data collection

Rigaku AFC10/Saturn724+ diffractometer
11602 measured reflections
3479 independent reflections
2673 reflections with *I* > 2σ(*I*)
*R*_int_ = 0.032

#### Refinement

*R*[*F* ^2^ > 2σ(*F* ^2^)] = 0.042
*wR*(*F* ^2^) = 0.114
*S* = 1.11
3479 reflections
177 parameters
H atoms treated by a mixture of independent and constrained refinement
Δρ_max_ = 0.21 e Å^−3^
Δρ_min_ = −0.18 e Å^−3^

### 

Data collection: *CrystalClear* (Rigaku, 2008 ▶); cell refinement: *CrystalClear*; data reduction: *CrystalClear*; program(s) used to solve structure: *SHELXS97* (Sheldrick, 2008 ▶); program(s) used to refine structure: *SHELXL97* (Sheldrick, 2008 ▶); molecular graphics: *SHELXTL* (Sheldrick, 2008 ▶); software used to prepare material for publication: *SHELXL97*.

## Supplementary Material

Crystal structure: contains datablock(s) I, global. DOI: 10.1107/S1600536812021575/nr2026sup1.cif

Structure factors: contains datablock(s) I. DOI: 10.1107/S1600536812021575/nr2026Isup2.hkl

Supplementary material file. DOI: 10.1107/S1600536812021575/nr2026Isup3.cml

Additional supplementary materials:  crystallographic information; 3D view; checkCIF report

**Fig. 1.**
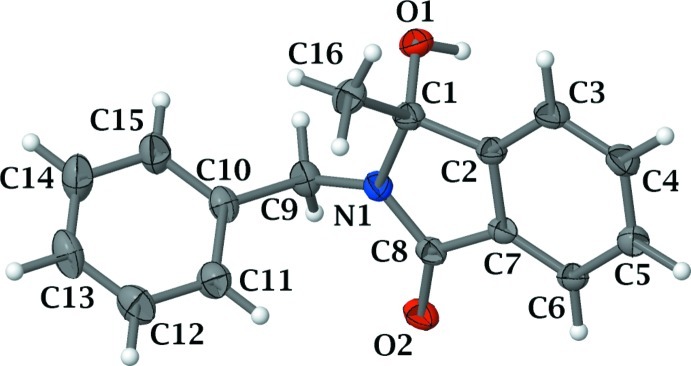
The molecular conformation of the title compound showing 50% probability displacement ellipsoids.

Enhanced figure: interactive version of Fig. 1

**Fig. 2.**
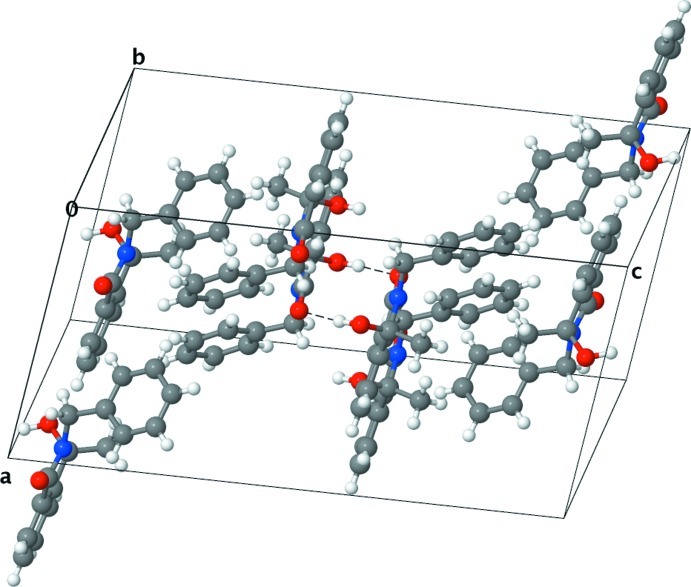
Molecular packing of the title compound.

Enhanced figure: interactive version of Fig. 2

## Figures and Tables

**Table 1:** Hydrogen-bond geometry (Å, °)

*D*—H⋯*A*	*D*—H	H⋯*A*	*D*⋯*A*	*D*—H⋯*A*
O1—H1*O*⋯O2^i^	0.960 (15)	1.836 (15)	2.7938 (14)	175.3 (12)

Symmetry code: (i) 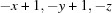.

## supplementary crystallographic information

### Comment

The title compound was obtained as a byproduct in the preparation of 2-benzyl-1,1,3,3-tetramethylisoindoline, an important intermediate in the synthesis of the radical 1,1,3,3-Tetramethylisoindolin-2-yloxyl (TMIO), which is used as spin probe and radical scavenger (Griffiths *et al.*, 1983). The title compound can be applied in the synthesis of heterocyclic amines through intramolecular amidoalkylation (Winn & Zaugg, 1968) and anionic ring-enlarging reaction (Katsuhiko *et al.*, 2006).

The molecular structure of the title compound is shown in Fig. 1 and there are some similiar stuctures reported before (Wang *et al.*, 2008; Orzeszko *et al.*, 1998; Liu *et al.*, 2009; Rosamilia *et al.*, 2002). In the molecule, the isoindol ring system is approximately planar [mean deviation = 0.0186 Å] and has a dihedral angle of 61.91 (4)° with the benzene ring. In the crystal (Fig. 2), molecules form centrosymmetric dimers *via* pairs of O—H···O hydrogen bonds (Table 1).

### Experimental

1.4 ml me thyl magnesium bromide solution (3 *M* in ether) was added to a 25 ml round-bottom flask filled with nitrogen and heated to 60 °C. A solution of *N*-benzylphthalimides (500 mg, 2.11 mmol) in toluene (15 ml) was added dropwise with stirring at a sufficient rate to maintain this temperature. When the addition was complete, the solution was heated to 110 °C and maintained at this temperature for 4 h. The reaction mixture was cooled to room temperature and petroleum was added. The mixture turned purple after stirring in air for 12 h. At the end, the mixture was filtered on celite and the filtrate obtained was dried, giving a precipitate which was separated by column chromatography on silica gel (eluent: ethyl acetate/petroleum ether, 1:3). The title compound was obtained as a colorless solid (123 mg, 23%) and evaporation of a solution in ethanol for 24 h afforded colorless single crystals suitable for X-ray diffraction.

### Refinement

The hydroxy H atom was obtained by difference Fourier synthesis and refined freely. All other H atoms were placed at calculated positions, with C—H = 0.95–0.98 Å. The *U*_iso_(H) values were constrained to be 1.5Ueq(C) for the methyl H atoms or 1.2Ueq(C) for the aromatic H atoms.

### Crystal data

**Table:**

C_16_H_15_NO_2_	*F*(000) = 1072
*M**_r_* = 253.29	*D*_x_ = 1.258 Mg m^−^^3^
Monoclinic, *C*2/*c*	Mo *K*α radiation, λ = 0.71073 Å
*a* = 11.093 (4) Å	Cell parameters from 4918 reflections
*b* = 11.604 (4) Å	θ = 2.6–29.1°
*c* = 21.226 (7) Å	µ = 0.08 mm^−^^1^
β = 101.777 (5)°	*T* = 153 K
*V* = 2674.7 (15) Å^3^	Block, colorless
*Z* = 8	0.47 × 0.34 × 0.23 mm

### Data collection

**Table:**

Rigaku AFC10/Saturn724+ diffractometer	2673 reflections with *I* > 2σ(*I*)
Radiation source: Rotating Anode	*R*_int_ = 0.032
Graphite monochromator	θ_max_ = 29.1°, θ_min_ = 2.6°
Detector resolution: 28.5714 pixels mm^-1^	*h* = −12→15
phi and ω scans	*k* = −15→15
11602 measured reflections	*l* = −28→28
3479 independent reflections	

### Refinement

**Table:**

Refinement on *F*^2^	Primary atom site location: structure-invariant direct methods
Least-squares matrix: full	Secondary atom site location: difference Fourier map
*R*[*F*^2^ > 2σ(*F*^2^)] = 0.042	Hydrogen site location: inferred from neighbouring sites
*wR*(*F*^2^) = 0.114	H atoms treated by a mixture of independent and constrained refinement
*S* = 1.11	*w* = 1/[σ^2^(*F*_o_^2^) + (0.056*P*)^2^ + 0.0264*P*] where *P* = (*F*_o_^2^ + 2*F*_c_^2^)/3
3479 reflections	(Δ/σ)_max_ < 0.001
177 parameters	Δρ_max_ = 0.21 e Å^−^^3^
0 restraints	Δρ_min_ = −0.18 e Å^−^^3^

### Special details

**Table:**

Geometry. All e.s.d.'s (except the e.s.d. in the dihedral angle between two l.s. planes) are estimated using the full covariance matrix. The cell e.s.d.'s are taken into account individually in the estimation of e.s.d.'s in distances, angles and torsion angles; correlations between e.s.d.'s in cell parameters are only used when they are defined by crystal symmetry. An approximate (isotropic) treatment of cell e.s.d.'s is used for estimating e.s.d.'s involving l.s. planes.
Refinement. Refinement of *F*^2^ against ALL reflections. The weighted *R*-factor *wR* and goodness of fit *S* are based on *F*^2^, conventional *R*-factors *R* are based on *F*, with *F* set to zero for negative *F*^2^. The threshold expression of *F*^2^ > σ(*F*^2^) is used only for calculating *R*-factors(gt) *etc*. and is not relevant to the choice of reflections for refinement. *R*-factors based on *F*^2^ are statistically about twice as large as those based on *F*, and *R*- factors based on ALL data will be even larger.

### Fractional atomic coordinates and isotropic or equivalent isotropic displacement parameters (Å^2^)

**Table:**

	*x*	*y*	*z*	*U*_iso_*/*U*_eq_	
O1	0.48310 (7)	0.73617 (7)	0.04556 (4)	0.0310 (2)	
O2	0.49294 (7)	0.35635 (7)	0.07278 (4)	0.0321 (2)	
N1	0.46720 (9)	0.54981 (8)	0.09038 (4)	0.0256 (2)	
C1	0.53806 (10)	0.65930 (9)	0.09452 (5)	0.0263 (2)	
C2	0.66237 (10)	0.61485 (9)	0.08633 (5)	0.0242 (2)	
C3	0.76900 (10)	0.67523 (10)	0.08427 (5)	0.0300 (3)	
H3	0.7719	0.7568	0.0880	0.036*	
C4	0.87197 (11)	0.61253 (10)	0.07655 (6)	0.0314 (3)	
H4	0.9459	0.6525	0.0746	0.038*	
C5	0.86960 (11)	0.49285 (10)	0.07166 (5)	0.0290 (3)	
H5	0.9418	0.4523	0.0672	0.035*	
C6	0.76204 (10)	0.43254 (9)	0.07334 (5)	0.0257 (3)	
H6	0.7589	0.3509	0.0699	0.031*	
C7	0.65951 (10)	0.49583 (9)	0.08021 (5)	0.0227 (2)	
C8	0.53351 (11)	0.45578 (9)	0.08088 (5)	0.0247 (2)	
C9	0.33419 (10)	0.54720 (10)	0.08518 (5)	0.0295 (3)	
H9A	0.3006	0.4809	0.0578	0.035*	
H9B	0.2989	0.6181	0.0628	0.035*	
C10	0.29071 (10)	0.53813 (10)	0.14795 (5)	0.0283 (3)	
C11	0.33512 (12)	0.45438 (11)	0.19347 (6)	0.0352 (3)	
H11	0.4002	0.4052	0.1873	0.042*	
C12	0.28499 (13)	0.44209 (12)	0.24794 (6)	0.0409 (3)	
H12	0.3159	0.3844	0.2788	0.049*	
C13	0.19061 (13)	0.51312 (13)	0.25754 (6)	0.0433 (3)	
H13	0.1557	0.5037	0.2945	0.052*	
C14	0.14733 (12)	0.59773 (14)	0.21325 (7)	0.0470 (4)	
H14	0.0832	0.6475	0.2200	0.056*	
C15	0.19722 (11)	0.61047 (12)	0.15877 (6)	0.0391 (3)	
H15	0.1671	0.6693	0.1285	0.047*	
C16	0.54084 (11)	0.71985 (10)	0.15828 (6)	0.0339 (3)	
H16A	0.5928	0.7887	0.1609	0.041*	
H16B	0.5745	0.6675	0.1938	0.041*	
H16C	0.4570	0.7423	0.1613	0.041*	
H1O	0.4958 (12)	0.7058 (13)	0.0054 (7)	0.059 (5)*	

### Atomic displacement parameters (Å^2^)

**Table:**

	*U*^11^	*U*^22^	*U*^33^	*U*^12^	*U*^13^	*U*^23^
O1	0.0387 (5)	0.0236 (4)	0.0314 (4)	0.0059 (3)	0.0092 (4)	0.0017 (3)
O2	0.0404 (5)	0.0228 (4)	0.0347 (5)	−0.0094 (3)	0.0114 (4)	−0.0022 (3)
N1	0.0259 (5)	0.0244 (5)	0.0278 (5)	−0.0027 (4)	0.0085 (4)	−0.0011 (4)
C1	0.0305 (6)	0.0207 (5)	0.0280 (6)	−0.0010 (4)	0.0069 (5)	−0.0011 (4)
C2	0.0286 (6)	0.0202 (5)	0.0240 (5)	−0.0011 (4)	0.0060 (4)	−0.0005 (4)
C3	0.0333 (6)	0.0208 (5)	0.0357 (6)	−0.0046 (5)	0.0063 (5)	−0.0001 (5)
C4	0.0281 (6)	0.0308 (6)	0.0355 (6)	−0.0052 (5)	0.0067 (5)	0.0040 (5)
C5	0.0290 (6)	0.0295 (6)	0.0290 (6)	0.0023 (5)	0.0073 (5)	0.0025 (5)
C6	0.0331 (7)	0.0202 (5)	0.0242 (5)	0.0007 (4)	0.0068 (5)	0.0009 (4)
C7	0.0287 (6)	0.0193 (5)	0.0202 (5)	−0.0024 (4)	0.0054 (4)	0.0007 (4)
C8	0.0322 (6)	0.0225 (5)	0.0201 (5)	−0.0037 (4)	0.0071 (4)	0.0001 (4)
C9	0.0266 (6)	0.0361 (6)	0.0260 (6)	−0.0022 (5)	0.0059 (5)	0.0008 (5)
C10	0.0238 (6)	0.0353 (6)	0.0256 (6)	−0.0062 (5)	0.0050 (4)	−0.0023 (5)
C11	0.0401 (7)	0.0376 (7)	0.0284 (6)	0.0017 (5)	0.0080 (5)	−0.0007 (5)
C12	0.0494 (9)	0.0469 (8)	0.0259 (6)	−0.0046 (6)	0.0069 (6)	0.0030 (5)
C13	0.0405 (8)	0.0640 (9)	0.0279 (6)	−0.0108 (7)	0.0132 (5)	−0.0030 (6)
C14	0.0350 (7)	0.0681 (10)	0.0419 (8)	0.0064 (7)	0.0174 (6)	0.0024 (7)
C15	0.0292 (7)	0.0531 (8)	0.0367 (7)	0.0031 (6)	0.0107 (5)	0.0069 (6)
C16	0.0387 (7)	0.0311 (6)	0.0325 (6)	0.0011 (5)	0.0086 (5)	−0.0084 (5)

### Geometric parameters (Å, º)

**Table:**

O1—C1	1.4102 (13)	C7—C8	1.4757 (16)
O1—H1O	0.960 (15)	C9—C10	1.5103 (16)
O2—C8	1.2377 (13)	C9—H9A	0.9900
N1—C8	1.3538 (14)	C9—H9B	0.9900
N1—C9	1.4573 (16)	C10—C11	1.3880 (17)
N1—C1	1.4871 (14)	C10—C15	1.3894 (17)
C1—C2	1.5151 (16)	C11—C12	1.3888 (18)
C1—C16	1.5196 (15)	C11—H11	0.9500
C2—C3	1.3831 (15)	C12—C13	1.380 (2)
C2—C7	1.3870 (15)	C12—H12	0.9500
C3—C4	1.3918 (16)	C13—C14	1.376 (2)
C3—H3	0.9500	C13—H13	0.9500
C4—C5	1.3925 (17)	C14—C15	1.3879 (18)
C4—H4	0.9500	C14—H14	0.9500
C5—C6	1.3900 (16)	C15—H15	0.9500
C5—H5	0.9500	C16—H16A	0.9800
C6—C7	1.3864 (15)	C16—H16B	0.9800
C6—H6	0.9500	C16—H16C	0.9800
			
C1—O1—H1O	107.6 (9)	N1—C9—C10	115.80 (10)
C8—N1—C9	122.99 (10)	N1—C9—H9A	108.3
C8—N1—C1	113.62 (9)	C10—C9—H9A	108.3
C9—N1—C1	122.48 (9)	N1—C9—H9B	108.3
O1—C1—N1	110.67 (9)	C10—C9—H9B	108.3
O1—C1—C2	113.42 (9)	H9A—C9—H9B	107.4
N1—C1—C2	100.65 (8)	C11—C10—C15	118.60 (11)
O1—C1—C16	106.91 (9)	C11—C10—C9	122.08 (11)
N1—C1—C16	111.22 (9)	C15—C10—C9	119.16 (11)
C2—C1—C16	113.96 (9)	C10—C11—C12	120.40 (12)
C3—C2—C7	120.32 (10)	C10—C11—H11	119.8
C3—C2—C1	129.45 (10)	C12—C11—H11	119.8
C7—C2—C1	110.23 (9)	C13—C12—C11	120.42 (13)
C2—C3—C4	117.82 (10)	C13—C12—H12	119.8
C2—C3—H3	121.1	C11—C12—H12	119.8
C4—C3—H3	121.1	C14—C13—C12	119.65 (13)
C3—C4—C5	121.78 (10)	C14—C13—H13	120.2
C3—C4—H4	119.1	C12—C13—H13	120.2
C5—C4—H4	119.1	C13—C14—C15	120.15 (13)
C6—C5—C4	120.23 (11)	C13—C14—H14	119.9
C6—C5—H5	119.9	C15—C14—H14	119.9
C4—C5—H5	119.9	C14—C15—C10	120.77 (13)
C7—C6—C5	117.57 (10)	C14—C15—H15	119.6
C7—C6—H6	121.2	C10—C15—H15	119.6
C5—C6—H6	121.2	C1—C16—H16A	109.5
C6—C7—C2	122.26 (10)	C1—C16—H16B	109.5
C6—C7—C8	129.28 (10)	H16A—C16—H16B	109.5
C2—C7—C8	108.44 (9)	C1—C16—H16C	109.5
O2—C8—N1	125.37 (11)	H16A—C16—H16C	109.5
O2—C8—C7	127.62 (10)	H16B—C16—H16C	109.5
N1—C8—C7	106.98 (9)		
			
C8—N1—C1—O1	119.71 (10)	C1—C2—C7—C8	2.63 (12)
C9—N1—C1—O1	−49.64 (13)	C9—N1—C8—O2	−7.00 (17)
C8—N1—C1—C2	−0.51 (11)	C1—N1—C8—O2	−176.29 (10)
C9—N1—C1—C2	−169.86 (9)	C9—N1—C8—C7	171.34 (9)
C8—N1—C1—C16	−121.59 (10)	C1—N1—C8—C7	2.05 (12)
C9—N1—C1—C16	69.06 (12)	C6—C7—C8—O2	−3.10 (19)
O1—C1—C2—C3	60.18 (15)	C2—C7—C8—O2	175.41 (11)
N1—C1—C2—C3	178.40 (11)	C6—C7—C8—N1	178.60 (10)
C16—C1—C2—C3	−62.48 (15)	C2—C7—C8—N1	−2.89 (12)
O1—C1—C2—C7	−119.60 (10)	C8—N1—C9—C10	99.09 (13)
N1—C1—C2—C7	−1.37 (11)	C1—N1—C9—C10	−92.55 (12)
C16—C1—C2—C7	117.75 (11)	N1—C9—C10—C11	−51.07 (15)
C7—C2—C3—C4	−0.60 (16)	N1—C9—C10—C15	133.51 (12)
C1—C2—C3—C4	179.64 (10)	C15—C10—C11—C12	1.30 (18)
C2—C3—C4—C5	−0.64 (17)	C9—C10—C11—C12	−174.15 (11)
C3—C4—C5—C6	1.07 (17)	C10—C11—C12—C13	−0.1 (2)
C4—C5—C6—C7	−0.23 (16)	C11—C12—C13—C14	−1.0 (2)
C5—C6—C7—C2	−1.03 (16)	C12—C13—C14—C15	0.9 (2)
C5—C6—C7—C8	177.31 (10)	C13—C14—C15—C10	0.3 (2)
C3—C2—C7—C6	1.47 (16)	C11—C10—C15—C14	−1.39 (19)
C1—C2—C7—C6	−178.73 (9)	C9—C10—C15—C14	174.19 (12)
C3—C2—C7—C8	−177.17 (9)		

### Hydrogen-bond geometry (Å, º)

**Table:**

*D*—H···*A*	*D*—H	H···*A*	*D*···*A*	*D*—H···*A*
O1—H1*O*···O2^i^	0.960 (15)	1.836 (15)	2.7938 (14)	175.3 (12)

Symmetry code: (i) −*x*+1, −*y*+1, −*z*.
